# 3,5-Bis(4-meth­oxy­phen­yl)-4,5-dihydro­isoxazole

**DOI:** 10.1107/S1600536811026833

**Published:** 2011-07-09

**Authors:** S. Samshuddin, Ray J. Butcher, Mehmet Akkurt, B. Narayana, H. S. Yathirajan

**Affiliations:** a Department of Studies in Chemistry, Mangalore University, Mangalagangotri 574 199, India; b Department of Chemistry, Howard University, 525 College Street NW, Washington, DC 20059, USA; c Department of Physics, Faculty of Sciences, Erciyes University, 38039 Kayseri, Turkey; d Department of Studies in Chemistry, University of Mysore, Manasagangotri, Mysore 570 006, India

## Abstract

In the title compound, C_17_H_17_NO_3_, the five-membered isoxazoline ring adopts an envelope conformation with the chiral C atom at the flap position and 0.133 (2) Å out of the mean plane formed by the other four atoms. The two benzene rings form dihedral angles of 6.05 (5) and 81.52 (5)° with the C—C—N—O plane of the isoxazoline ring. The crystal structure is stabilized by weak C—H⋯O hydrogen bonds and C—H⋯π inter­actions.

## Related literature

For medical uses of isoxazole derivatives, see: Sperry & Wright (2005 ▶). For their biological activity, see; Boyd (1991 ▶); Lang & Lin (1984 ▶). For related structures, see; Baktır *et al.* (2011*a* ▶,*b* ▶); Chopra *et al.* (2006 ▶); Dardouri *et al.* (2010 ▶); Fun *et al.* (2010*a* ▶,*b* ▶); Guo *et al.* (2006 ▶); Jasinski *et al.* (2010 ▶); Ko *et al.* (2011 ▶); Samshuddin *et al.* (2010 ▶). For ring puckering and asymmetry parameters, see: Cremer & Pople (1975 ▶); Nardelli (1983 ▶).
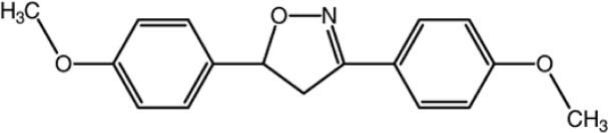


## Experimental

### 

#### Crystal data

C_17_H_17_NO_3_
*M* *_r_* = 283.32
Orthorhombic, 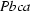

*a* = 10.5071 (7) Å
*b* = 8.4023 (5) Å
*c* = 32.6662 (19) Å
*V* = 2883.9 (3) Å^3^
*Z* = 8
Mo *K*α radiation
μ = 0.09 mm^−1^
*T* = 295 K
0.42 × 0.36 × 0.16 mm

#### Data collection

Oxford Diffraction Xcalibur Ruby Gemini diffractometer
Absorption correction: multi-scan (*CrysAlis PRO*; Oxford Diffraction, 2007 ▶) *T*_min_ = 0.967, *T*_max_ = 1.000
13952 measured reflections
4821 independent reflections
2738 reflections with i > 2σ(i)
*R*_int_ = 0.046

#### Refinement

*R*[*F* ^2^ > 2σ(*F* ^2^)] = 0.075
*wR*(*F* ^2^) = 0.156
*S* = 1.06
4821 reflections
192 parameters
H-atom parameters constrained
Δρ_max_ = 0.23 e Å^−3^
Δρ_min_ = −0.17 e Å^−3^

### 

Data collection: *CrysAlis PRO* (Oxford Diffraction, 2007 ▶); cell refinement: *CrysAlis PRO*; data reduction: *CrysAlis RED* (Oxford Diffraction, 2007 ▶); program(s) used to solve structure: *SHELXS97* (Sheldrick, 2008 ▶); program(s) used to refine structure: *SHELXL97* (Sheldrick, 2008 ▶); molecular graphics: *ORTEP-3 for Windows* (Farrugia, 1997 ▶); software used to prepare material for publication: *WinGX* (Farrugia, 1999 ▶) and *PLATON* (Spek, 2009 ▶).

## Supplementary Material

Crystal structure: contains datablock(s) global, I. DOI: 10.1107/S1600536811026833/im2301sup1.cif

Structure factors: contains datablock(s) I. DOI: 10.1107/S1600536811026833/im2301Isup2.hkl

Supplementary material file. DOI: 10.1107/S1600536811026833/im2301Isup3.cml

Additional supplementary materials:  crystallographic information; 3D view; checkCIF report

## Figures and Tables

**Table 1:** Hydrogen-bond geometry (Å, °) *Cg*2 and *Cg*3 are the centroids of the C1*A*–C6*A* and C1*B*–C6*B* benzene rings, respectively.

*D*—H⋯*A*	*D*—H	H⋯*A*	*D*⋯*A*	*D*—H⋯*A*
C6*A*—H6*AA*⋯O1^i^	0.93	2.52	3.324 (2)	145
C1—H1*A*⋯*Cg*3^ii^	0.98	2.62	3.590 (2)	170
C6*B*—H6*BA*⋯*Cg*3^iii^	0.93	3.00	3.724 (2)	136
C7*B*—H7*BC*⋯*Cg*2^iv^	0.96	2.83	3.541 (3)	132

Symmetry codes: (i) 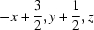; (ii) 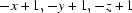; (iii) 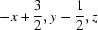; (iv) 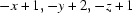.

**Fig. 1.**
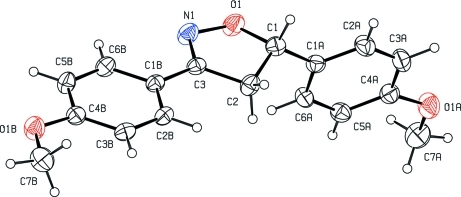
Molecular structure of the title compound with ellipsoids drawn at the 30% probability level and H atoms as spheres of arbitrary radius.

**Fig. 2.**
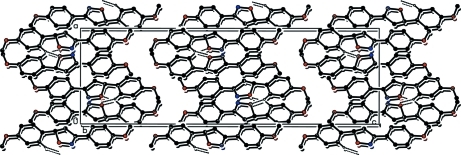
Packing diagram of the title molecule, showing C—H···O interactions, viewed down the b axis. Hydrogen atoms not involved in H-bonding have been omitted for clarity.

## supplementary crystallographic information

### Comment

Chalcones are convenient intermediates for the synthesis of various biodynamic cyclic derivatives such as isoxazoline, pyrazoline, benzodiazepine and cyclohexenone derivatives (Samshuddin *et al.*, 2010; Fun *et al.*, 2010*a*,*b*; Jasinski *et al.*, 2010; Baktır *et al.*, 2011*a*,*b*). Isoxazole derivatives represent a unique class of nitrogen- and oxygen-containing five-membered heterocycles. These are the components in a variety of natural products and medicinally useful compounds (Sperry *et al.*, 2005). Isoxazole derivatives bearing different substituents are known to have various biological activities in pharmaceutical and agricultural areas (Lang & Lin, 1984; Boyd, 1991).

The crystal structures of some 4,5-dihydroisoxazole derivatives *viz*., 5-(4-fluoro-3-phenoxyphenyl)-3-(4-methylphenyl)-4,5-dihydroisoxazole (Chopra *et al.*, 2006), 5-(1*H*-indol-3-yl)-3-(4-methylphenyl)-4,5-dihydroisoxazoline (Guo *et al.*, 2006), 1,5-dimethyl-3-[(3-phenyl-4,5-dihydro-1,2-oxazol-5-yl)methyl]-1*H*-1,5- benzodiazepine-2,4(3*H*,5*H*)-dione (Dardouri *et al.*, 2010) and (*S*)-[5-methyl-3-(3-methylthiophen-2-yl)-4,5-dihydroisoxazol-5-yl] methanol solvate (Ko *et al.*, 2011) have been reported. In view of the importance of isoxazoles and in continuation of our work on synthesis of various derivatives of chalcones, the title compound (I) was prepared and its crystal structure is reported.

As shown in Fig. 1, the 4,5-dihydroisoxazole ring (O1/N1/C1–C3) of the title compound (I) has an envelope conformation with the chiral C1 atom at the flap position, displaced from the mean plane by -0.133 (2) Å; the puckering parameters (Cremer & Pople, 1975) are Q(2) = 0.2156 (17) Å, φ(2) = 319.7 (5)°. Furthermore, one (O1B/C1B–C7B) of the methoxyphenyl ring systems makes a dihedral angle of 6.05 (5)°, whereas the other one (O1A/C1A–C7A) is almost orthogonal to the plane formed by the four atoms O1, N1, C2 and C3 of the five-membered isoxazoline ring, the dihedral angle being 81.52 (5)° (Nardelli, 1983). The dihedral angle between the methoxyphenyl ring systems (O1A/C1A–C7A and O1B/C1B–C7B) is 76.56 (4)°.

In the crystal structure, molecules are linked by weak C6A—H6AA···O1 (Table 1, Fig. 2) hydrogen bonds, and are further consolidated by C–H···π interactions (Table 1, Cg2 and Cg3 are the centroids of the C1A–C6A and C1B–C6B benzene rings, respectively).

### Experimental

A solution of (2*E*)-1,3-bis(4-methoxyphenyl)prop-2-en-1-one (2.68 g, 0.01 mol) and hydroxylamine hydrochloride (0.695 g, 0.01 mol) in 25 ml ethanol containing 3 ml of 10% sodium hydroxide solution was refluxed for 12 h. After cooling the mixture was poured into 50 ml ice-cold water. The resulting precipitate was collected by filtration and purified by recrystallization from ethanol. The single-crystal was grown from 2-propanol by slow evaporation of the solvent (yield: 59%; (m.p.: 407 K).

### Refinement

H atoms were located geometrically (aromatic C—H = 0.93 Å, methyl C—H = 0.96 Å, methylene C—H = 0.97 Å and methine C—H = 0.98 Å) and refined using the riding model approximation with fixed isotropic displacement parameters: *U*_iso_(H) = 1.5 *U*_eq_(methyl-C) and *U*_iso_(H) = 1.2 *U*_eq_(other C atoms).

### Crystal data

**Table:**

C_17_H_17_NO_3_	*F*(000) = 1200
*M**_r_* = 283.32	*D*_x_ = 1.305 Mg m^−^^3^
Orthorhombic, *P**b**c**a*	Mo *K*α radiation, λ = 0.71073 Å
Hall symbol: -P 2ac 2ab	Cell parameters from 2593 reflections
*a* = 10.5071 (7) Å	θ = 5.2–32.6°
*b* = 8.4023 (5) Å	µ = 0.09 mm^−^^1^
*c* = 32.6662 (19) Å	*T* = 295 K
*V* = 2883.9 (3) Å^3^	Triangular plate, colourless
*Z* = 8	0.42 × 0.36 × 0.16 mm

### Data collection

**Table:**

Oxford Diffraction Xcalibur Ruby Gemini diffractometer	4821 independent reflections
Radiation source: Enhance (Mo) X-ray Source	2738 reflections with i > 2σ(i)
graphite	*R*_int_ = 0.046
Detector resolution: 10.5081 pixels mm^-1^	θ_max_ = 32.7°, θ_min_ = 5.2°
ω scans	*h* = −12→15
Absorption correction: multi-scan (*CrysAlis PRO*; Oxford Diffraction, 2007)	*k* = −10→12
*T*_min_ = 0.967, *T*_max_ = 1.000	*l* = −29→46
13952 measured reflections	

### Refinement

**Table:**

Refinement on *F*^2^	Primary atom site location: structure-invariant direct methods
Least-squares matrix: full	Secondary atom site location: difference Fourier map
*R*[*F*^2^ > 2σ(*F*^2^)] = 0.075	Hydrogen site location: inferred from neighbouring sites
*wR*(*F*^2^) = 0.156	H-atom parameters constrained
*S* = 1.06	*w* = 1/[σ^2^(*F*_o_^2^) + (0.0468*P*)^2^ + 0.4739*P*] where *P* = (*F*_o_^2^ + 2*F*_c_^2^)/3
4821 reflections	(Δ/σ)_max_ = 0.001
192 parameters	Δρ_max_ = 0.23 e Å^−^^3^
0 restraints	Δρ_min_ = −0.17 e Å^−^^3^

### Special details

**Table:**

Geometry. Bond distances, angles *etc*. have been calculated using the rounded fractional coordinates. All su's are estimated from the variances of the (full) variance-covariance matrix. The cell e.s.d.'s are taken into account in the estimation of distances, angles and torsion angles
Refinement. Refinement on *F*^2^ for ALL reflections except those flagged by the user for potential systematic errors. Weighted *R*-factors *wR* and all goodnesses of fit *S* are based on *F*^2^, conventional *R*-factors *R* are based on *F*, with *F* set to zero for negative *F*^2^. The observed criterion of *F*^2^ > σ(*F*^2^) is used only for calculating -*R*-factor-obs *etc*. and is not relevant to the choice of reflections for refinement. *R*-factors based on *F*^2^ are statistically about twice as large as those based on *F*, and *R*-factors based on ALL data will be even larger.

### Fractional atomic coordinates and isotropic or equivalent isotropic displacement parameters (Å^2^)

**Table:**

	*x*	*y*	*z*	*U*_iso_*/*U*_eq_	
O1	0.70804 (13)	0.51832 (16)	0.43065 (4)	0.0604 (5)	
O1A	0.63367 (15)	0.77980 (18)	0.25241 (4)	0.0702 (5)	
O1B	0.62961 (12)	0.83077 (16)	0.65053 (4)	0.0603 (5)	
N1	0.72185 (15)	0.54460 (19)	0.47317 (4)	0.0554 (5)	
C1	0.58593 (17)	0.5848 (2)	0.41763 (5)	0.0480 (6)	
C1A	0.59901 (16)	0.6424 (2)	0.37429 (5)	0.0424 (5)	
C1B	0.63231 (14)	0.69607 (18)	0.52798 (5)	0.0381 (5)	
C2	0.55553 (17)	0.7045 (2)	0.45099 (5)	0.0487 (6)	
C2A	0.51887 (17)	0.5861 (2)	0.34384 (6)	0.0534 (6)	
C2B	0.53722 (15)	0.7969 (2)	0.54218 (5)	0.0420 (5)	
C3	0.63763 (14)	0.64427 (19)	0.48509 (5)	0.0392 (5)	
C3A	0.53294 (18)	0.6342 (2)	0.30376 (6)	0.0588 (7)	
C3B	0.53190 (15)	0.8445 (2)	0.58282 (5)	0.0440 (5)	
C4A	0.62771 (17)	0.7403 (2)	0.29317 (5)	0.0476 (6)	
C4B	0.62455 (15)	0.7929 (2)	0.60983 (5)	0.0435 (5)	
C5A	0.70837 (17)	0.7983 (2)	0.32303 (5)	0.0485 (6)	
C5B	0.71995 (16)	0.6916 (2)	0.59621 (5)	0.0502 (6)	
C6A	0.69329 (16)	0.7490 (2)	0.36319 (5)	0.0472 (5)	
C6B	0.72320 (15)	0.6433 (2)	0.55609 (5)	0.0459 (6)	
C7A	0.7369 (2)	0.8744 (3)	0.23880 (6)	0.0751 (9)	
C7B	0.53076 (19)	0.9273 (3)	0.66684 (6)	0.0651 (8)	
H2AA	0.45450	0.51470	0.35060	0.0640*	
H1A	0.52180	0.50020	0.41830	0.0580*	
H3AA	0.47830	0.59510	0.28370	0.0710*	
H2A	0.57900	0.81170	0.44300	0.0580*	
H2B	0.46610	0.70220	0.45830	0.0580*	
H5AA	0.77240	0.87000	0.31620	0.0580*	
H6AA	0.74780	0.78840	0.38320	0.0570*	
H7AA	0.73380	0.88390	0.20950	0.1130*	
H7AB	0.81560	0.82520	0.24670	0.1130*	
H7AC	0.73120	0.97830	0.25090	0.1130*	
H2BA	0.47560	0.83330	0.52400	0.0500*	
H3BA	0.46660	0.91050	0.59180	0.0530*	
H5BA	0.78200	0.65630	0.61440	0.0600*	
H6BA	0.78700	0.57420	0.54750	0.0550*	
H7BA	0.54280	0.93930	0.69580	0.0980*	
H7BB	0.45000	0.87780	0.66170	0.0980*	
H7BC	0.53280	1.03010	0.65400	0.0980*	

### Atomic displacement parameters (Å^2^)

**Table:**

	*U*^11^	*U*^22^	*U*^33^	*U*^12^	*U*^13^	*U*^23^
O1	0.0765 (9)	0.0589 (8)	0.0457 (7)	0.0266 (7)	0.0012 (6)	−0.0037 (6)
O1A	0.0859 (10)	0.0801 (10)	0.0445 (8)	−0.0125 (8)	−0.0025 (7)	0.0053 (7)
O1B	0.0668 (8)	0.0680 (9)	0.0461 (7)	0.0106 (7)	−0.0018 (6)	−0.0049 (6)
N1	0.0640 (10)	0.0565 (10)	0.0458 (8)	0.0209 (8)	0.0000 (7)	0.0006 (7)
C1	0.0512 (10)	0.0445 (10)	0.0484 (10)	−0.0028 (8)	−0.0005 (8)	0.0025 (7)
C1A	0.0459 (9)	0.0381 (9)	0.0433 (9)	0.0013 (7)	0.0011 (7)	−0.0018 (7)
C1B	0.0353 (8)	0.0358 (8)	0.0431 (9)	−0.0013 (7)	0.0019 (7)	0.0057 (6)
C2	0.0467 (9)	0.0551 (11)	0.0442 (9)	0.0109 (8)	0.0026 (7)	0.0033 (8)
C2A	0.0503 (10)	0.0533 (11)	0.0565 (11)	−0.0148 (9)	−0.0028 (8)	0.0009 (8)
C2B	0.0387 (8)	0.0410 (9)	0.0463 (9)	0.0046 (7)	−0.0007 (7)	0.0084 (7)
C3	0.0371 (8)	0.0354 (8)	0.0452 (9)	−0.0005 (7)	0.0028 (7)	0.0051 (6)
C3A	0.0607 (12)	0.0630 (12)	0.0528 (11)	−0.0120 (10)	−0.0144 (9)	−0.0034 (9)
C3B	0.0428 (9)	0.0387 (9)	0.0504 (10)	0.0047 (7)	0.0067 (7)	0.0044 (7)
C4A	0.0534 (10)	0.0480 (10)	0.0413 (9)	0.0038 (9)	0.0011 (7)	−0.0012 (7)
C4B	0.0462 (9)	0.0427 (9)	0.0417 (9)	−0.0033 (8)	0.0020 (7)	0.0031 (7)
C5A	0.0493 (10)	0.0458 (10)	0.0505 (10)	−0.0062 (8)	0.0029 (8)	0.0004 (7)
C5B	0.0439 (10)	0.0568 (11)	0.0500 (10)	0.0083 (8)	−0.0081 (7)	0.0044 (8)
C6A	0.0482 (9)	0.0480 (10)	0.0454 (9)	−0.0055 (8)	−0.0050 (7)	−0.0055 (7)
C6B	0.0384 (9)	0.0483 (10)	0.0511 (10)	0.0087 (8)	0.0017 (7)	0.0024 (8)
C7A	0.0860 (16)	0.0809 (16)	0.0584 (13)	−0.0076 (13)	0.0103 (11)	0.0144 (11)
C7B	0.0696 (13)	0.0732 (14)	0.0524 (12)	0.0015 (11)	0.0105 (9)	−0.0100 (10)

### Geometric parameters (Å, °)

**Table:**

O1—N1	1.4139 (19)	C4B—C5B	1.388 (2)
O1—C1	1.463 (2)	C5A—C6A	1.385 (2)
O1A—C4A	1.374 (2)	C5B—C6B	1.372 (2)
O1A—C7A	1.416 (3)	C1—H1A	0.9800
O1B—C4B	1.368 (2)	C2—H2A	0.9700
O1B—C7B	1.421 (3)	C2—H2B	0.9700
N1—C3	1.279 (2)	C2A—H2AA	0.9300
C1—C1A	1.503 (2)	C2B—H2BA	0.9300
C1—C2	1.517 (2)	C3A—H3AA	0.9300
C1A—C2A	1.386 (2)	C3B—H3BA	0.9300
C1A—C6A	1.384 (2)	C5A—H5AA	0.9300
C1B—C2B	1.390 (2)	C5B—H5BA	0.9300
C1B—C3	1.468 (2)	C6A—H6AA	0.9300
C1B—C6B	1.397 (2)	C6B—H6BA	0.9300
C2—C3	1.497 (2)	C7A—H7AA	0.9600
C2A—C3A	1.378 (3)	C7A—H7AB	0.9600
C2B—C3B	1.388 (2)	C7A—H7AC	0.9600
C3A—C4A	1.381 (3)	C7B—H7BA	0.9600
C3B—C4B	1.384 (2)	C7B—H7BB	0.9600
C4A—C5A	1.381 (2)	C7B—H7BC	0.9600
			
N1—O1—C1	108.43 (13)	C1—C2—H2A	112.00
C4A—O1A—C7A	118.35 (15)	C1—C2—H2B	112.00
C4B—O1B—C7B	117.94 (14)	C3—C2—H2A	112.00
O1—N1—C3	109.29 (14)	C3—C2—H2B	112.00
O1—C1—C1A	108.47 (14)	H2A—C2—H2B	109.00
O1—C1—C2	103.24 (13)	C1A—C2A—H2AA	119.00
C1A—C1—C2	118.86 (14)	C3A—C2A—H2AA	119.00
C1—C1A—C2A	120.70 (15)	C1B—C2B—H2BA	119.00
C1—C1A—C6A	121.39 (15)	C3B—C2B—H2BA	119.00
C2A—C1A—C6A	117.88 (15)	C2A—C3A—H3AA	120.00
C2B—C1B—C3	121.78 (14)	C4A—C3A—H3AA	120.00
C2B—C1B—C6B	117.75 (15)	C2B—C3B—H3BA	120.00
C3—C1B—C6B	120.47 (14)	C4B—C3B—H3BA	120.00
C1—C2—C3	100.90 (13)	C4A—C5A—H5AA	120.00
C1A—C2A—C3A	121.09 (16)	C6A—C5A—H5AA	120.00
C1B—C2B—C3B	121.60 (15)	C4B—C5B—H5BA	120.00
N1—C3—C1B	120.73 (14)	C6B—C5B—H5BA	120.00
N1—C3—C2	113.17 (14)	C1A—C6A—H6AA	119.00
C1B—C3—C2	126.01 (14)	C5A—C6A—H6AA	119.00
C2A—C3A—C4A	120.32 (17)	C1B—C6B—H6BA	120.00
C2B—C3B—C4B	119.44 (15)	C5B—C6B—H6BA	119.00
O1A—C4A—C3A	115.58 (16)	O1A—C7A—H7AA	109.00
O1A—C4A—C5A	124.85 (16)	O1A—C7A—H7AB	109.00
C3A—C4A—C5A	119.57 (16)	O1A—C7A—H7AC	109.00
O1B—C4B—C3B	125.04 (15)	H7AA—C7A—H7AB	109.00
O1B—C4B—C5B	115.21 (14)	H7AA—C7A—H7AC	109.00
C3B—C4B—C5B	119.72 (15)	H7AB—C7A—H7AC	110.00
C4A—C5A—C6A	119.56 (16)	O1B—C7B—H7BA	109.00
C4B—C5B—C6B	120.35 (15)	O1B—C7B—H7BB	109.00
C1A—C6A—C5A	121.58 (16)	O1B—C7B—H7BC	109.00
C1B—C6B—C5B	121.12 (15)	H7BA—C7B—H7BB	110.00
O1—C1—H1A	109.00	H7BA—C7B—H7BC	109.00
C1A—C1—H1A	109.00	H7BB—C7B—H7BC	109.00
C2—C1—H1A	109.00		
			
C1—O1—N1—C3	−12.91 (18)	C2B—C1B—C3—N1	−176.15 (16)
N1—O1—C1—C1A	148.33 (13)	C2B—C1B—C3—C2	7.6 (2)
N1—O1—C1—C2	21.37 (16)	C6B—C1B—C3—N1	3.2 (2)
C7A—O1A—C4A—C3A	−173.31 (17)	C6B—C1B—C3—C2	−173.08 (15)
C7A—O1A—C4A—C5A	6.5 (3)	C2B—C1B—C6B—C5B	−1.1 (2)
C7B—O1B—C4B—C3B	1.7 (3)	C3—C1B—C6B—C5B	179.54 (15)
C7B—O1B—C4B—C5B	−176.75 (16)	C1—C2—C3—N1	14.78 (19)
O1—N1—C3—C1B	−178.53 (14)	C1—C2—C3—C1B	−168.71 (15)
O1—N1—C3—C2	−1.81 (19)	C1A—C2A—C3A—C4A	−0.1 (3)
O1—C1—C1A—C2A	123.44 (17)	C1B—C2B—C3B—C4B	1.2 (2)
O1—C1—C1A—C6A	−54.6 (2)	C2A—C3A—C4A—O1A	179.75 (16)
C2—C1—C1A—C2A	−119.19 (18)	C2A—C3A—C4A—C5A	−0.1 (3)
C2—C1—C1A—C6A	62.8 (2)	C2B—C3B—C4B—O1B	−179.72 (15)
O1—C1—C2—C3	−20.68 (16)	C2B—C3B—C4B—C5B	−1.4 (2)
C1A—C1—C2—C3	−140.76 (15)	O1A—C4A—C5A—C6A	−179.71 (16)
C1—C1A—C2A—C3A	−177.79 (16)	C3A—C4A—C5A—C6A	0.1 (3)
C6A—C1A—C2A—C3A	0.3 (3)	O1B—C4B—C5B—C6B	178.89 (15)
C1—C1A—C6A—C5A	177.80 (16)	C3B—C4B—C5B—C6B	0.4 (3)
C2A—C1A—C6A—C5A	−0.2 (3)	C4A—C5A—C6A—C1A	0.0 (3)
C3—C1B—C2B—C3B	179.44 (15)	C4B—C5B—C6B—C1B	0.9 (3)
C6B—C1B—C2B—C3B	0.1 (2)		

### Hydrogen-bond geometry (Å, °)

**Table:**

Cg2 and Cg3 are the centroids of the C1A–C6A and C1B–C6B benzene rings, respectively.

**Table:**

*D*—H···*A*	*D*—H	H···*A*	*D*···*A*	*D*—H···*A*
C6A—H6AA···O1^i^	0.93	2.52	3.324 (2)	145
C1—H1A···Cg3^ii^	0.98	2.62	3.590 (2)	170
C6B—H6BA···Cg3^iii^	0.93	3.00	3.724 (2)	136
C7B—H7BC···Cg2^iv^	0.96	2.83	3.541 (3)	132

Symmetry codes: (i) −*x*+3/2, *y*+1/2, *z*; (ii) −*x*+1, −*y*+1, −*z*+1; (iii) −*x*+3/2, *y*−1/2, *z*; (iv) −*x*+1, −*y*+2, −*z*+1.

## References

[bb1] Baktır, Z., Akkurt, M., Samshuddin, S., Narayana, B. & Yathirajan, H. S. (2011*a*). *Acta Cryst.* E**67**, o1262–o1263. 10.1107/S1600536811015455 PMC3089231 21754550

[bb2] Baktır, Z., Akkurt, M., Samshuddin, S., Narayana, B. & Yathirajan, H. S. (2011*b*). *Acta Cryst.* E**67**, o1292–o1293. 10.1107/S160053681101587X PMC3120319 21754699

[bb3] Boyd, G. V. (1991). *Prog. Heterocyl. Chem.* **3**, 166–185.

[bb4] Chopra, D., Mohan, T. P. & Vishalakshi, B. (2006). *Acta Cryst.* E**62**, o3547–o3548. 10.1107/S010827010602378X 16954636

[bb5] Cremer, D. & Pople, J. A. (1975). *J. Am. Chem. Soc.* **97**, 1354–1358.

[bb6] Dardouri, R., Kandri Rodi, Y., Saffon, N., El Ammari, L. & Essassi, E. M. (2010). *Acta Cryst.* E**66**, o2983. 10.1107/S1600536810042972 PMC3009296 21589149

[bb7] Farrugia, L. J. (1997). *J. Appl. Cryst.* **30**, 565.

[bb8] Farrugia, L. J. (1999). *J. Appl. Cryst.* **32**, 837–838.

[bb9] Fun, H.-K., Hemamalini, M., Samshuddin, S., Narayana, B. & Yathirajan, H. S. (2010*a*). *Acta Cryst.* E**66**, o582–o583. 10.1107/S1600536810004435 PMC2983722 21580348

[bb10] Fun, H.-K., Hemamalini, M., Samshuddin, S., Narayana, B. & Yathirajan, H. S. (2010*b*). *Acta Cryst.* E**66**, o864–o865. 10.1107/S1600536810009414 PMC2983895 21580687

[bb11] Guo, X.-H., Qi, X.-X. & Chang, J.-B. (2006). *Acta Cryst.* E**62**, o374–o375.

[bb12] Jasinski, J. P., Guild, C. J., Samshuddin, S., Narayana, B. & Yathirajan, H. S. (2010). *Acta Cryst.* E**66**, o1948–o1949. 10.1107/S1600536810026036 PMC3007318 21588274

[bb13] Ko, Y. K., Ryu, J. W., Koo, D. W., Woo, J. C. & Kim, C.-H. (2011). *Acta Cryst.* E**67**, o1040. 10.1107/S1600536811011639 PMC3089274 21754367

[bb14] Lang, A. & Lin, Y. (1984). *Comprehensive Heterocyclic Chemistry*, Vol. 6, edited by A. R. Katritzky, pp. 1–130. Oxford: Pergamon Press.

[bb15] Nardelli, M. (1983). *Acta Cryst.* C**39**, 1141–1142.

[bb16] Oxford Diffraction (2007). *CrysAlis PRO* and *CrysAlis RED* Oxford Diffraction Ltd, Abingdon, England.

[bb17] Samshuddin, S., Narayana, B., Yathirajan, H. S., Safwan, A. P. & Tiekink, E. R. T. (2010). *Acta Cryst.* E**66**, o1279–o1280. 10.1107/S1600536810015795 PMC2979444 21579379

[bb18] Sheldrick, G. M. (2008). *Acta Cryst.* A**64**, 112–122. 10.1107/S0108767307043930 18156677

[bb19] Spek, A. L. (2009). *Acta Cryst.* D**65**, 148–155. 10.1107/S090744490804362X PMC2631630 19171970

[bb20] Sperry, J. & Wright, D. (2005). *Curr. Opin. Drug Discov. Dev.* **8**, 723–740. 16312148

